# Frequency of orthodontic extraction

**DOI:** 10.1590/2177-6709.21.1.054-059.oar

**Published:** 2016

**Authors:** Camila de S. Dardengo, Luciana Q. P. Fernandes, Jonas Capelli

**Affiliations:** 1 MSc, Universidade Estadual do Rio de Janeiro (UERJ), Department of Orthodontics, Rio de Janeiro, Brazil; 2 Orthodontist, Universidade Estadual do Rio de Janeiro (UERJ), Department of Orthodontics, Rio de Janeiro, Brazil; 3 Coordinator, Post-graduation Program (Doctorate), Universidade do Estado do Rio de Janeiro (UERJ), Department of Orthodontics, Rio de Janeiro, Brazil

**Keywords:** Tooth extraction, Epidemiology, Orthodontics

## Abstract

**Introduction:**

The option of dental extraction for orthodontic purposes has been debated for more than 100 years, including periods when it was widely used in treatment, including the present, during which other methods are used to avoid dental extractions. The objective was to analyze the frequency of tooth extraction treatment performed between 1980 and 2011 at the Orthodontic Clinic of Universidade Estadual do Rio de Janeiro (UERJ).

**Material and Methods:**

The clinical records of 1484 patients undergoing orthodontic treatment were evaluated. The frequency of extractions was evaluated with regard to sex, Angle's classification, the different combinations of extractions and the period when orthodontic treatment began. Chi-square test was used to determine correlations between variables, while the chi-square test for trends was used to assess the frequency of extractions over the years.

**Results:**

There was a reduction of approximately 20% in the frequency of cases treated with tooth extraction over the last 32 years. The most frequently extracted teeth were first premolars. Patients with Class I malocclusion showed fewer extractions, while Class II patients underwent a higher number of extraction treatment. There were no statistically significant differences with regard to sex.

**Conclusion:**

New features introduced into the orthodontic clinic and new esthetic concepts contributed to reducing the number of cases treated with dental extractions. However, dental extractions for orthodontic purposes are still well indicated in certain cases.

## Abstract (pt)

**Introdução:**

a extração dentária com finalidade ortodôntica é debatida há mais de 100 anos, oscilando entre períodos nos quais foi amplamente utilizada e períodos, como os atuais, onde outros métodos são utilizados visando evitar as extrações dentárias.

**Objetivo:**

analisar a frequência de extrações dentárias, entre os anos de 1980 e 2011, na Clínica de Especialização em Ortodontia da Faculdade de Odontologia da Universidade do Estado do Rio de Janeiro.

**Métodos:**

foram avaliadas as documentações ortodônticas de 1484 pacientes. A frequência de extrações foi avaliada em relação ao sexo, à classificação de Angle, às combinações de extração e ao período de início do tratamento ortodôntico. Utilizou-se o teste qui-quadrado para verificar a correlação entre as variáveis, e o teste qui-quadrado para tendência para avaliar a frequência de extração ao longo dos anos.

**Resultados e Conclusão:**

houve uma redução de aproximadamente 20% na frequência de tratamentos ortodônticos com extrações dentárias ao longo de 32 anos. Os dentes mais extraídos foram os quatro primeiros pré-molares. Os pacientes portadores da má oclusão de Classe I apresentaram menos tratamentos com extrações, enquanto os pacientes com Classe II apresentaram maior número de tratamentos com extração. Não houve diferenças estatisticamente significativas com relação ao sexo.

## INTRODUCTION

At the beginning of the twentieth century, when Orthodontics became a science, treatment plans were based on the premise that extraction destroys the possibility of ideal occlusion or ideal esthetics. For this reason, they were condemned by Edward Hartley Angle and his followers.^1^

One of Angle's most important opponents was Calvin Case who defended dental extractions for correcting facial deformities due to excessive dental or maxillary protrusion. According to Case, extraction was necessary in 3% of cases that presented Class I malocclusion, in 5% of Class II cases and nearly 0% of Class III cases. Thus, considering the incidence of these malocclusions, only 6 to 7% of treated cases required extractions.^2^^,^ 3

In 1930, after Angle died, one of his followers, Charles Tweed, who had evaluated cases that had been previously treated without extractions, decided to retreat several cases that presented relapses. After analyzing the cases treated in accordance with Angle's philosophy, he noticed that 80% of his patients did not achieve stability, facial esthetics, periodontal health or functional objectives. For this reason, Tweed defended extractions as a method for achieving facial harmony and providing greater post-treatment stability.^4^ This finding caused a revolution in orthodontic thinking, and, by the end of the 1940s, extractions were reintroduced.^5^

Between 1950 and 1960, dental extractions for orthodontic purposes became common in the United States. Approximately 50% of patients underwent orthodontic treatment with teeth extractions, usually first premolars.^6^

After 1960, with greater acceptance of Begg's technique, orthodontists who did not employ the Edgewise philosophy adopted this new technique and incorporated extractions into their orthodontic planning. At that time, dental extractions reached their peak and thereafter began to decrease considerably.^5^

As noted, the decision to extract teeth for orthodontic purposes has been debated for more than a hundred years. Currently, the criteria that guide orthodontic extractions go beyond cast analysis and the position of teeth in the bone base. The decision for tooth extraction, especially in borderline cases, requires dental, facial and skeletal evaluations to obtain an accurate diagnosis and effective treatment plan. Patient's cooperation, facial profile and skeletal age, the presence of dental asymmetry and anteroposterior relations, as well as the presence of pathology, are determining factors in the decision-making involving dental extraction in Orthodontics.^7^^,^ 8 ^,^ 9

Concerns regarding esthetic facial aging can be added to the list of factors that strongly influence orthodontic planning nowadays,^10^ although there are some studies which affirm that extraction treatment does not adversely impact soft tissue profile changes over time^11^ and does not change patient's facial height.^12^ Moreover, the improvement of bonding in Orthodontics and the introduction of various techniques, such as interproximal reduction, thermoplastic aligners, functional appliances, self-ligated brackets and temporary anchorage devices, also influence orthodontic planning.^10^

Even though these resources often promote expansion and space gain in the arches, extractions remain included in orthodontic plans that seek to improve facial appearance and achieve stable results.^10^Several papers in the literature have suggested first premolars as the major indication for extraction for orthodontic purposes.^6^^,^ 13 ^-^ 17 The choice of these teeth is justified because of their proximity to anterior and posterior teeth and because they occupy an intermediate position in the arch, which facilitates correction of crowding, dentoalveolar protrusion and midline deviations.^17^

Based on the observed fluctuation in the frequency of orthodontic extractions over the years, this study aimed to analyze, from the point of view of the frequency of dental extractions, cases treated in the Orthodontics Department of Universidade Estadual do Rio de Janeiro, from 1980 to 2011. The frequency of extractions was evaluated with regard to sex, malocclusion classification according to Angle, different extraction combinations and the period when orthodontic treatment began.

## MATERIAL AND METHODS

In this study, 1484 records from patients submitted to comprehensive orthodontic treatment between 1980 and 2011 at the Orthodontic Clinic of Universidade Estadual do Rio de Janeiro (UERJ) were used. This research was submitted and approved by the university Ethics Committee (942.974).

Sample inclusion criteria were as follows:

» Clinical charts that allowed the definition of treatment plan with regard to orthodontic extractions,
» Presence of all permanent teeth in the oral cavity or under intraosseous development. Third molars were not taken into consideration.

Patients with inconclusive records, i.e., patients for whom it was not possible to determine whether the plan consisted of extractions, were excluded from the sample. In addition, patients who had tooth agenesis or mutilation were also excluded because treatment plan could have changed, considering the necessity of extractions for orthodontic reasons.

For data registration, Microsoft Excel^TM^ 2007 software was used. The spreadsheet can be seen in Table 1.

**Table 1:** - Example of the spreadsheet used for data registration.

**Registration number**	**Sex**	**Start date**	**Angle's classification**	**Extraction**	**Extracted teeth**				
			**C I**	**C II D 1**	**C II D 2**	**C III**	**Yes**	**No**	
									
									
									
									

After data collection, the frequency of extractions was evaluated with regard to sex, Angle's classification, different extraction combinations and the period when orthodontic treatment began.

To determine whether there were correlations between variables, chi-square test was used, with significance set at 5%. Results were divided into six groups (1980 to 1985, 1986 to 1990, 1991 to 1996, 1997 to 2001, 2002 to 2006 and 2007 to 2011), and chi-square analysis for trend was performed to test for statistical significance of changes in extraction rates over time.

## RESULTS

The sample consisted of 829 female patients (55.9%) and 655 male patients (44.1%). Regarding the frequency of orthodontic treatment with extractions, the sample showed 680 cases treated with extraction (45.8%) and 804 cases treated without extraction (54.2%). Considering Angle's classification, there were 735 patients with Class I (49.5%), 559 patients with Class II, Division 1 (37.7%), 137 patients with Class III (9.2%) and 53 patients with Class II, Division 2 malocclusion (3.6%).

**Figure 1:**
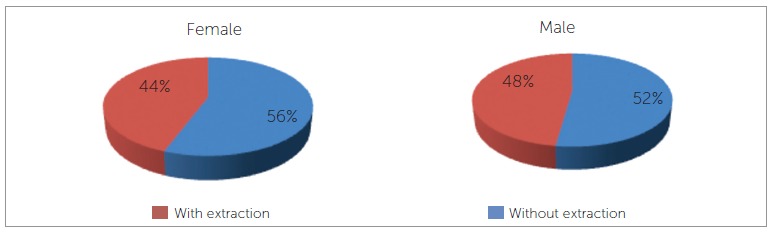
- Frequency of treatment with and without extraction in both males and females.

The frequencies of extraction in female patients and male patients were compared, and no significant differences were found between them ( *p* = 0.1773). In both cases, treatment without extractions was more frequent (Fig 1).

**Figure 2:**
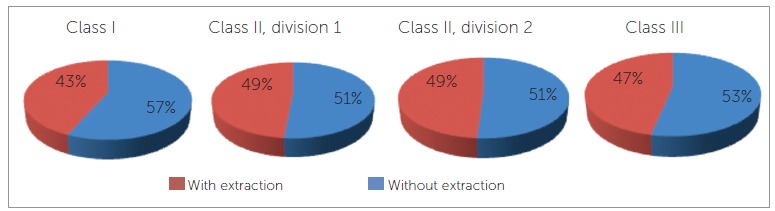
- Frequency of treatment with and without extraction according to Angle's classification.

The frequency of extraction with regard to Angle's classification was analyzed (Fig 2), and no significant differences were found among classes ( *p* = 0.3090). Treatment of patients with Class I malocclusion showed the lowest frequency of extraction, whereas the greatest frequency was observed in patients with Class II malocclusion.

Among treatment modalities performed with extractions, the frequency of different extraction combinations was compared according to Angle's classification (Table 2). Extraction of four first premolars was the most frequent combination, followed by extraction of only maxillary first premolars.

**Table 2:** - The most frequent extraction combinations in relation to the total sample and Angle's classification.

	**1^st^ most frequent combination**	**2^nd^ most frequent combination**	**3^rd^ most frequent combination**
Total	14, 24, 34 and 44 (48.8%)	14 and 24 (14.5%)	14, 24, 35 and 45 (4.1%)
Class I	14, 24, 34 and 44 (66.8%)	1 lower incisor (6%)	14 and 24 (3.8%)
Class II, Division 1	14, 24, 34 and 44 (36.2%)	14 and 24 (20.3%)	14, 24, 35 and 45 (6.3%)
Class II, Division 2	14 and 24 (34.6%)	14, 24, 34 and 44 (15.4%)	1 lower incisor (7.7%)
Class III	14 and 24 (31.3%)	14, 24, 34 and 44 (23.4%)	15, 25, 34 and 44 (14%)

**Figure 3:**
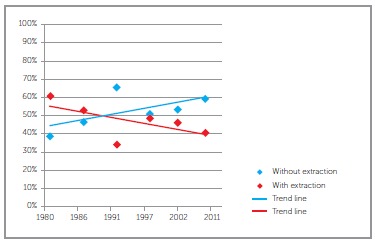
- Frequency of treatment with and without extraction in both males and females in the whole sample.

The present study also analyzed the frequency of extractions for orthodontic purposes from 1980 to 2011. By dividing this period into six groups, the frequency of extraction was observed over time (Fig 3), and the results revealed significant differences among these groups ( *p*= 0.01732).

## DISCUSSION

Few studies in the literature have evaluated periods longer than 20 years in order to study the frequency of dental extractions for orthodontic purposes. The present study found a reduction in the number of cases treated with extractions from 1980 to 2011. Proffit's study,^5^ which evaluated a period of 40 years, Moreira's study,^16^ which evaluated a period of 30 years, and Janson's study,^18^ which evaluated a period of 35 years, also found a reduction in orthodontic extractions over time. 

Changes in esthetic standards over time and an increasing variety of resources available to the orthodontist to treat a malocclusion, such as expander systems, distalizers devices, functional and orthopedic appliances, temporary anchorage devices and an increased frequency of interproximal reduction can be pointed out as the primary reasons for the decrease in the number of extractions in orthodontic treatment, as observed by Proffit^5^ and in the present study. The advent of techniques using bonded brackets, instead of banding of all teeth, also contributed to the reduction in tooth extractions, as observed in previous studies through the 1970s. Moreover, according to Proffit,^5^ long-term studies that showed no stability expected in patients treated with extractions also contributed to the decline in the frequency of treatment with extractions.

An analysis of the graph presented in Figure 3revealed that the frequency of cases treated with extractions decreased from 61.1% in 1980 to 40.8% in 2011. A detailed analysis of this graph reveals the greatest reduction in extraction treatment in the period from 1991 to 1996. This finding can most likely be explained by the sample having been treated at a university where some of the new treatment techniques are experimented in order to assess their effectiveness and outcomes. During this period, a greater number of resources was likely used for space gain necessary for alignment and correction of dental irregularities. The characteristics inherent to treatment conducted at universities were also cited by another research.^17^

In line with other studies,^6^^,^ 13 ^-^ 17 ^,^ 19 first premolars were the most commonly extracted teeth. It is believed that this finding is due to the location of these elements in the dental arch, which favors the correction of midline deviations and space problems in the incisor region.^13^^,^ 17

The choice of teeth to be extracted should consider their position in the arch in addition to other factors, such as cavities, changes in development, endodontic treatment, extensive restorations and/or restorations of poor quality and ectopic location.^13^

The analysis of the frequency of different tooth extraction ratings, according to Angle's classification, revealed no statistically significant differences, as observed in another study.^16^ However, in the current study, patients with Class I malocclusion presented the lowest frequency of extractions (43%), while in Moreira's study^16^these patients had the greatest frequency of treatment with extractions (68.6%).^16^

Although Calvin Case was opposed to Angle's ideas and proposed treatment with extractions in the early twentieth century, its indications were still far fewer than those observed nowadays. In his study,^2^extractions were hardly ever indicated in cases of Class III malocclusion, whereas in the present study, extractions in Class III patients occurred in 47% of cases. In addition, in these patients, maxillary first premolars were the most frequently indicated teeth to be extracted (31.3%). This finding can most likely be explained by the consolidation of surgical techniques for treatment of Class III malocclusion.

Although no statistically significant differences were identified, this study found a higher frequency of extractions in male patients (48%), while in female patients extractions were performed in 44% of cases. Other studies have corroborated these findings,^16^^,^ 20 but Peck and Peck's study^9^ observed a higher frequency of extractions in female patients (44%), while only 39% of male patients were treated with extractions. This reduction in tooth extraction in female patients, which is observed in more recent studies, has most likely been justified by the growing concern with esthetics in this population. Extractions followed by retraction of anterior teeth result in a reduction of profile convexity and deepening of facial furrows, which are condemned by current esthetic standards.

## CONCLUSION

The number of orthodontic cases involving extractions has decreased over time. The frequency of tooth extraction observed in this study, over a period of 32 years, decreased by approximately 20%.

The teeth most often extracted were four first premolars, followed by the option of extracting only maxillary first premolars.

Analysis of malocclusion revealed that the greatest number of extractions was observed in patients with Class II malocclusion, whereas patients with Class I malocclusion presented the lowest number of cases with extractions. There were no statistically significant differences with regard to sex.

## References

[1] Angle EH (1907). Treatment of malocclusion of the teeth.

[2] Case CS (1912). The question of extraction in orthodontia. Dent Cosmos.

[3] Case CS (1913). The question of extraction: an answer to Dr. Ferris' discussion. Dent Cosmos.

[4] Tweed CH (1944). Indications for the extraction of teeth in orthodontic procedure. Am J Orthod.

[5] Proffit WR (1994). Forty-year review of extraction frequencies at a university orthodontic clinic. Angle Orthod.

[6] Salzmann JA (1965). An evaluation of extraction in orthodontics. Am J Orthod.

[7] Strang RHW, Thompson WM (1958). A text-book of orthodontia.

[8] Ruellas ACO, Ruellas RMO, Romano FL, Pithon MM, Santos RL (2010). Tooth extraction in orthodontics: an evaluation of diagnostic elements. Dental Press J Orthod.

[9] Peck S, Peck H (1979). Frequency of tooth extraction in orthodontic treatment. Am J Orthod.

[10] Graber LW, Vanarsdall RL, Vig KWL (2011). The decision-making process in orthodontics in Orthodontics: current principles and techniques.

[11] Rathod AB, Araujo E, Vaden JL, Behrents RG, Oliver DR (2015). Extraction vs no treatment: Long-term facial profile changes. Am J Orthod Dentofacial Orthop.

[12] Zafarmand AH, Zafarmand MM (2015). Premolar extraction in orthodontics: Does it have any effect on patient's facial height?. J Int Soc Prev Community Dent.

[13] Brandt S, Safirstein GR (1975). Different extractions for different malocclusions. Am J Orthod.

[14] Bradbury AJ (1985). A current view on patterns of extraction therapy in British health service orthodontics. Br Dent J.

[15] Hooper JD (1967). Orthodontics as a public service: the Wessex survey. Dent Pract Dent Rec.

[16] Moreira TC, Mucha NA (1997). A freqüência de exodontias em tratamentos ortodônticos realizados na Clínica do Curso de Mestrado em Ortodontia da Faculdade de Odontologia da UFRJ. Ortodon Gaúch.

[17] Weintraub JA, Vig PS, Brown C, Kowalski CJ (1989). The prevalence of orthodontic extractions. Am J Orthod Dentofacial Orthop.

[18] Janson G, Maria FR, Bombonatti R (2014). Frequency evaluation of different extraction protocols in orthodontic treatment during 35 years. Prog Orthod.

[19] Gaya C, Capelli JJr, Oliveira RS, Silva ACP (1999). Frequências de extração em pacientes submetidos a tratamento ortodôntico. Rev SBO.

[20] Gaya C, Capelli Jr, Oliveira RS, Silva ACP (2003). Estudo da frequência das extrações dentárias do tratamento ortodôntico. Rev SBO.

